# Editorial: Insect Olfactory Proteins (From Gene Identification to Functional Characterization)

**DOI:** 10.3389/fphys.2019.01313

**Published:** 2019-10-18

**Authors:** Peng He, Nicolas Durand, Shuang-Lin Dong

**Affiliations:** ^1^State Key Laboratory Breeding Base of Green Pesticide and Agricultural Bioengineering, Key Laboratory of Green Pesticide and Agricultural Bioengineering, Ministry of Education, Guizhou University, Guiyang, China; ^2^FRE CNRS 3498, Ecologie et Dynamique des Systèmes Anthropisés, Université de Picardie Jules Verne, Amiens, France; ^3^Education Ministry Key Laboratory of Integrated Management of Crop Disease and Pests, College of Plant Protection, Nanjing Agricultural University, Nanjing, China

**Keywords:** odorant-binding proteins, chemosensory proteins, odorant receptors, ionotropic receptors, odorant-degrading enzymes

Olfaction is essential for the survival and reproduction of many insects. Their extremely sophisticated and sensitive olfactory system helps them accomplish key behaviors, such as seeking food resources, avoiding predators, locating mate partners, and selecting egg-laying sites. The involved molecular actors, the olfactory proteins, play crucial roles in the responses triggered by external chemical stimuli. Classification includes receptor proteins, the odorant receptors (ORs) and the ionotropic receptors (IRs), and perireceptor proteins, the odorant binding proteins (OBPs), chemosensory proteins (CSPs), sensory neuron membrane proteins (SNMPs) and odorant degrading enzymes (ODEs). Olfactory proteins are expressed in olfactory sensilla, hair-like structures predominantly located on insect antennae. Sensilla house the olfactory receptor neurons (ORNs), whose dendrites bath in a lymph. Recent advances in bioinformatics and biological techniques have enabled the identification of numerous olfactory-related genes and unveiled their functions. However, our knowledge of the molecular mechanisms of the insect olfactory system is still very limited. This Research Topic is presenting the identification and the molecular and functional characterization of insect olfactory proteins in diverse insect species. One third of articles are dedicated to the moths (Lepidoptera), reflecting the outstanding interest in the chemical ecology of these diverse crop or forest pests. The other articles are treating olfactory proteins of representatives of hemipteran, coleopteran, dipteran, and orthopteran pests or disease vectors, respectively. In addition, three articles focus on parasitoid or pollinating hymenopterans and two articles present comparative studies between species from at least two insect orders.

## Modern Sequencing Technologies Facilitate the Identification and Expression Analysis of Novel Olfactory Genes and Proteins

In the last decade, high throughput sequencing of transcriptomics and proteomics has become widely used to identify the large repertoire of olfactory genes in numerous insect species, and to investigate their expression between various physiological states and tissues. Here, Jin et al. analyzed antennal transcriptomes from the oriental fruit fly *Bactrocera dorsalis*, corresponding to different maturity and mating status, and identified 43 ORs and 21 IRs. Many of them are strongly regulated by mating or egg-laying, reflecting that these olfactory genes could have close relationships with these crucial behaviors (Jin et al.). Similarly, in the chive gnat *Bradysia odoriphaga*, analysis of antennal and body adult transcriptomes led to the identification of 49 OBPs and 5 CSPs. 22 OBPs and 3 CSPs are preferentially expressed in the antennae, including 9 male enriched OBPs which are relevant candidates for sex pheromone component binding and transport (Zhao et al.). In the beet armyworm *Spodoptera exigua*, Zhang et al. analyzed transcripts expressed in the chemosensory organs of adults and identified 64 ORs, 22 IRs, 24 OBPs and 19 CSPs. In another study, 53 ORs and 4 IRs have been identified in the antennae of the citrus long-horned beetle *Anoplophora chinensis* by transcriptomic analysis (Sun et al.). In the vetch aphid *Megoura viciae*, 10 OBPs were identified in the antennal transcriptome (Bruno et al.). A great diversity of putative ODEs was also unveiled in the antennal transcriptomes of two moths, 33 glutathione-S-transferases (GSTs) in *Spodoptera littoralis* (Durand et al.) and 35 carboxylesterases (CXEs) in *Ectropis obliqua* (Sun et al.). To study the evolution of insect chemoreception, Yuvaraj et al. analyzed the antennal expressed genes and compared the olfactory repertoires of two basal Lepidoptera species and one species of its sister group Trichoptera. Combined phylogenetic analysis suggests that the pheromone receptors (PRs) and the pheromone binding proteins (PBPs) have evolved in parallel with the transition of sex pheromone types in Lepidoptera, while other chemoreceptor subfamilies show a broader taxonomic occurrence than hitherto acknowledged (Yuvaraj et al.). Wen et al. sequenced the antennal transcriptomes of two closely related weevils *Eucryptorrhynchus scrobiculatus* and *E. brandti*, reared on the same host plants although on different parts. Total numbers of olfactory genes identified in the two species were similar, 111 (49 ORs, 17 IRs, 31 OBPs, 11 CSPs, and 3 SNMPs) in *E. scrobiculatus*, and 112 (45 ORs, 25 IRs, 28 OBPs 11 CSPs, and 3 SNMPs) in *E. brandti*, however species-specific olfactory genes were highlighted, with a possible role in the recognition of specific volatiles from different plant parts. Olfactory genes were identified in the sibling moth species, *Ectropis grisescens* and *E. obliqua*, including 59 ORs, 24 IRs, 40 OBPs and 30 CSPs, from *E. grisescens* male antennae (Li et al.) and 52 ORs and 36 OBPs from *E. obliqua* (Li et al.). Fan et al. paired two sequencing techniques, next generation sequencing [NGS and single-molecule real-time sequencing (SMRT)] to obtain full-length olfactory genes in the parasitoid *Aphidius gifuensis*, including 66 ORs, 25 IRs, 16 OBPs, and 12 CSPs. Among them, 25 proteins could be potentially involved in aphid alarm pheromone E-β-farnesene detection. Zhang et al. compared the antennal transcriptomes between virgin and mated female adults of the moth *Dendrolimus punctatus*, and identified new olfactory-related genes including 8 ORs and 5 IRs. In addition, a subset of olfactory proteins is up-regulated after mating, indicating a putative association with oviposition site seeking behavior (Zhang et al.). Das De et al. conducted a comparative analysis of prior and post-blood meal groups in the mosquito *Anopheles culicifacies*, and unraveled several ORs and OBPs that could drive blood feeding associated behaviors in this vector.

Olfactory genes are mainly identified in insect antennae but they could also be highlighted in other chemosensory organs, such as palps and proboscis. Here, Guo et al. analyzed second generation sequencing data of the labial palps and proboscis of the moth *Helicoverpa armigera*, and unveiled a vast number of olfactory genes including 4 ORs, 6 IRs, 39 OBPs, 26 CSPs, and 2 SNMPs. Similarly, 11 ORs and 16 OBPs have been identified from antennae, maxillary and labial palps transcriptome of the locust *Locusta migratoria*, including four ORs (*OR12, OR13, OR14*, and *OR18)* and *OBP8*, which are expressed in higher levels in palps than in antennae (Li et al.). In addition, a limited number of olfactory genes have been identified in the leg transcriptome of the mirid bug *Adelphocoris lineolatus* (Sun et al.), with only 8 OBPs. Proteomics also lead to the identification and the expression analysis at the protein level. Iovinella et al. adopted a proteomic approach to investigate the expression levels of olfactory proteins (OBPs, CSPs, and ODEs) in honeybees among different castes, tasks and ages, highlighting major differences between queens and workers. In addition, based on isobaric tags for relative and absolute quantitation (ITRAQ) comparative proteomic analysis, Song et al. unveiled that two OBPs and three CSPs are differently expressed between alate and apterous morphs of the pea aphid *Acyrthosiphon pisum*.

## Molecular and Functional Characterization of Odorant Receptors

ORs are seven-transmembrane receptors located on the dendrites of ORNs and activated by OBP/CSP-odorant complexes or odorants only. ORs can be divided into OR co-receptors (*Orcos*) and ligand-specific ORs (ORx), which interact with each other to form an ORx-ORco complex to generate a ligand-gated cation channel. *Orcos* are well-conserved among insect species and *Orco* knock-out insects are anosmic, indicating *Orcos* as promising pest management targets. In this Research Topic, Wang et al. compared and analyzed *Orco* genes from five sibling mirid bug species: *Apolygus lucorum, Lygus pratensis, A. lineolatus, A. suturalis*, and *A. fasciaticollis*. The five *Orco* genes shared high deduced amino acid identities and are well-conserved. However, at genome level, these five *Orco* genes present significantly different exon-intron structures, especially on the insertion sites and length of introns, suggesting variation in their evolution rates (Wang et al.). Pheromone receptors (PRs) are an ORx subtype generally located in trichoid sensilla and activated by sex pheromone components. PRs have been deorphanized in many species, especially in Lepidoptera. In this Research Topic, Liu et al. verified the correspondence between PRs and a series of sex pheromone components of the Asian corn borer *Ostrinia furnacalis*. The results obtained *in vitro* with the *Xenopus* oocyte expression system are in line with the *in vivo* electrophysiological analysis of four types of trichoid sensilla (Liu et al.). The functional characterization methods for PRs or other ORxs are multiple and could produce variability, which may result in somewhat different conclusions. Wang et al. compared two functional assays, *in vivo* transgenic fly system and *in vitro Xenopus* oocyte expression system, using three PR clades (*HarmOR6, OR13*, and *OR16*) from three phylogenetically close noctuid moths, *H. armigera, H. assulta*, and *Heliothis virescens*. Results from the two methods seem consistent, with nevertheless an increased sensitivity of the *in vitro* system. However, the active sex pheromone components of OR6 obtained by the two methods are different in *H. armigera* and *H. assulta*, and therefore, other techniques such as genome editing combined with behavior tests are needed to further verify the precise functions of olfactory receptors in the future (Wang et al.).

Compared to PRs, broadly tuned ORxs are complex to deorphanize, because each ORx is tuned to a variety of odorants, while one odorant could bound to multiple ORxs. Furthermore, non-PR ORxs in general are highly divergent in amino acid sequence among insect species. In this Research Topic, Liu et al. showcased a comprehensive method to deorphanize one OR, *BdorOR88a*, expressed in adult males of *B. dorsalis*. First, a comparison of gene expression in the antennal transcriptomes of methyl eugenol (ME)-exposed and control insects highlighted that two OR genes, *BdorOR63a-1* and *BdorOR88a*, are up-regulated after ME exposure. Then a complementary *in vitro* functional study demonstrated that only BdorOR88a/Orco robustly responded to ME. Finally, behavioral experiments with *BdorOR88a* knock-down male flies revealed a reduced attraction of these insects to ME (Liu et al.). In another study, Li et al. first determined the active odorants that produced increased electrophysiological responses with maxillary and labial palps as compared with antennae in *L. migratoria*. Then, palp transcriptomes were analyzed and four palp-enriched ORs (*LmigOR12, OR13, OR14*, and *OR18)* were identified. Finally, RNA interference (RNAi) combined to electroantennogram recordings indicated that *OR12* was responsible for detection of three aldehyde odorants (E,E)-2,4-heptadienal, hexanal, and E-2-hexenal (Li et al.). These approaches could be used to deorphanize more broadly tuned ORxs. In addition, the cell-based expression system combined with Ca^2+^ level investigation could be used to set up a high throughput and rapid screening of vast potential odorant candidates.

## Odorant Carrying Proteins Potentially Involved in Pheromone and Plant Volatile Detection

OBPs and CSPs abundantly expressed in insect sensillar lymph are considered as putative carriers of odorants to the chemosensory receptors anchored in the dendritic membrane of ORNs. Pheromone binding proteins (PBPs) constitute a sub-group of OBPs participating in sex pheromone detection. Here, two PBPs, *CpunPBP2* and *CpunPBP5*, enriched in the antennae of the adult male of the yellow peach moth *Conogethes punctiferalis* have been functionally characterized. Recombinant proteins presented *in vitro* extremely high binding abilities with the two sex pheromone components compared to a pheromone analog and a panel of plant volatiles. Moreover, activity of mutated proteins indicate that several amino acid residues are potentially involved in sex pheromone binding (Ge et al.). In the Chagas disease vector *Rhodnius prolixus*, Oliveira et al. focused on two male antennae-specific PBPs, *RproOBP26* and *RproOBP27* and performed RNAi experiments suggesting that *RproOBP27* is involved in sex pheromone detection. In the eastern honey bee *Apis cerana*, a PBP(AcerOBP11) displays *in vitro* strong affinities with the main queen mandibular pheromone compounds, the alarm pheromone and worker pheromone components. Experiments with mutated PBPs demonstrated that two residues (Ile97 and Ile140) play crucial roles in binding with various bee pheromones (Song et al.).

In this Research Topic, the important role of OBPs for aggregation pheromone recognition has also been demonstrated. In adults of the red palm weevil *Rhynchophorus ferrugineus*, Antony et al. first identified four antennal-enriched or antennal-specific OBPs, and then determined that *RferOBP1768* plays a major role in detection of the aggregation pheromone by RNAi experiments. Similarly, Guo et al. uncovered that OBPs could be involved in aggregation pheromone detection. Seventeen OBPs were identified in the genome of *L. migratoria*, two of which, *LmigOBP2* and *LmigOBP4*, are up-regulated during gregarization and down-regulated during solitarization. Subsequently, they performed RNAi and behavioral experiments, and proposed that *LmigOBP4* is the sole OBP that potentially participates in aggregation pheromone detection in *L. migratoria* (Guo et al.).

Additional studies in this Research Topic indicated that certain OBPs could present broad ligand affinity, suggesting their multiple roles in chemosensation. Ma et al. identified *EoblOBP6* in *E. obliqua*, as an OBP dominantly expressed in the antennae and legs of adult insects, and presenting strong affinities with plant volatiles (such as benzaldehyde, nerolidol, α-farnesene) and with the aversive bitter alkaloid berberine, suggesting roles in both olfaction and gustation. In another study, *GmolOBP7*, an OBP from the oriental fruit moth *Grapholita molesta* is highly expressed in antennae and wings of both sexes. *In vitro* ligand binding assays and *in vivo* RNAi experiments suggested a dual role of GmolOBP7 in detection of both sex pheromone components and host plant volatiles (Chen et al.).

Sensilla expression patterns of OBPs in the insect antennae can provide valuable information about their putative roles and likely interplays among OBP partners within a sensillum. In the desert locust *Schistocerca gregaria*, Jiang et al. first demonstrated that the OBP repertoire is divided into four major phylogenetic clades, then characterized the specific sensilla expression patterns of representatives from each OBP clade. OBPs of subclade I-A are expressed in both trichoid and basiconic sensilla, while members of subclade II-A are restricted to coeloconic sensilla. OBPs of III-A, III-B, and I-B are exclusively found in chaetic sensilla, with a specific OBP of I-B being also expressed in coeloconic sensilla. The atypical OBP subtype from subclade IV-A is expressed in a subpopulation of coeloconic sensilla, and lastly, the plus-C type-B OBP subtypes from subclade IV-B are expressed in all four antennal sensillum types (trichoid, basiconic, chaetic, and coeloconic). Furthermore, a subset of OBPs, such as *SgreOBP1, 2, 5, 6, 10*, and *14*, are co-localized in the same sensilla and could interact as partners to be active (Jiang et al.; Jiang et al.). Some OBPs are also located in other organs besides antennae, including non-chemosensory organs, indicating their multiple roles apart from olfaction. In the vetch aphid *M. viciae*, Bruno et al. first described the distribution of three types of sensilla (trichoid, coeloconic, and placoid) on antennae, mouthparts, legs and cauda. Then functional hypotheses were determined based on the distribution profiles of 5 OBPs in these sensilla by immunolocalization (Bruno et al.).

CSP display greater sequence conservation but wider tissue expression patterns compared to OBPs, implying multiple putative functions. In this Research Topic, a few CSPs are associated with insect olfaction or gustation. Li et al. first successfully identified a nymph antennae-enriched CSP from the sycamore lace bug *Corythucha ciliata, CcilCSP2*, by screening the tissue expression pattern of 15 CSPs. Then recombinant CcilCSP2 was functionally characterized *in vitro*. CcilCSP2 binds with high affinity to the alarm pheromone geraniol and to the repellent phenyl benzoate (Li et al.). Another two studies further characterized the olfactory function of CSPs using RNAi experiments followed with electrophysiological or behavioral tests. In CSP knock-down insects, electrophysiological responses toward plant volatiles are diminished, and host plant location is altered. Li et al. identified a CSP, *DarmCSP2*, preferentially expressed in the antennae of the Chinese white pine beetle *Dendroctonus armandi*. The *in vitro* ligand-binding assays showed that the sex pheromone components and terpene host plant volatiles are potential active ligands of DarmCSP2. Further electrophysiological tests showed that the response of *DarmCSP2* knock-down individuals to (+)-α-pinene, (+)-3-carene, (+)-β-pinene, myrcene, (–)-β-pinene, and (+)-camphene declined steeply compared to control insects (Li et al.). In the brown planthopper *Nilaparvata lugens*, Waris et al. showed that NlugCSP8 displayed wide binding abilities with rice plant volatiles such as nerolidol and hexanal in ligand binding experiments. Furthermore, the knock-down insects displayed the significant loss of attraction to the set of plant odorants (Waris et al.).

Functional studies of odorant carrying proteins as well as other olfactory proteins, could be of great importance for the improvement of a variety of pest control methods. One such application, known as “reverse chemical ecology,” utilize the target olfactory proteins to screen for high affinity chemicals, which are in turn used to develop more effective agents for environmentally friendly pest management. In this Research Topic, two antennal-enriched OBPs of the dark black chafer beetle *Holotrichia parallela, HparOBP20*, and *HparOBP49*, bind with high affinity to the green leaf volatile 3-hexenyl acetate, which triggers high electrophysiological and behavioral activity for adult insects. Introduction of this compound into the field traps significantly increases male catches when combined with the sex pheromone (Ju et al.).

## Identification of Putative Odorant-Degrading Enzymes

ODEs include multiple enzyme families expressed in the sensillar lymph and likely involved in the fast inactivation of odorants to keep the olfactory system sensitive. In this Research Topic, Sun et al. suggested ODEs could convert ester odorants to inactive corresponding alcohols in *E. obliqua*. Expression analysis revealed that 12 CXEs are enriched in insect antennae. Complementary localization experiments determined that the signals of *EbolCXE7* and *EbolCXE13* engulfed not only in trichoid and basiconic olfactory sensilla but also in putative gustatory styloconic sensilla, indicating a putative dual role in both olfaction and gustation (Sun et al.). A second study determined the tissue expression profiles of 33 GSTs identified in *S. littoralis* antennae by transcriptomic approach, and highlighted four *SlitGSTs* (*SlitGSTd2, e9, e15*, and *MGST1-3*) dominantly expressed in olfactory organs and potentially acting as ODEs (Durand et al.). However, the enzymatic activities toward odorants for these ODE candidates need to be assessed. As a general mechanism, the “rapid” inactivation of odorants by ODEs needs more functional studies with different ODE families and insect species, as there are only few evidences that support this mechanism. As reflected in this Research Topic, ODEs draw much less attention from researchers than odorant receptors (ORs) and odorant carriers (OBPs and CSPs). However, major advances on the understanding of the function of these antennal enzymes could be expected in the future.

## Prospects, Challenges, and Potential Applications on Pest Management

Although numerous olfactory-related genes have been identified and functionally characterized, downstream applications in pest management are very limited. Nevertheless, achievements obtained in gene identification and functional clarification are critical for application aspects of pest and vector control. In this context, mosquitoes are major targets since they transmit various deadly viruses and other diseases to human populations around the globe. These vectors rely mainly on human emanations to seek and distinguish their hosts. In this Research Topic, Sparks et al. proposed feasible approaches for mosquito management by focusing on chemosensory receptors (especially ORs and IRs), including CRISPR-CAS9-mediated alterations of these receptors. Co-receptors like *Orco* and *Irco* (*IR8a, IR25a*, and *IR76b*) should be the primary candidate genes for mosquito management because they are well-conserved among all mosquito species (Sparks et al.). However, there are still several barriers on pest management by using chemosensory genes. Venthur and Zhou reviewed the progress on chemosensory gene characterization (ORs and OBPs) and compared the feasibility on pest management between ORs and OBPs by highlighting their advantages and drawbacks. OBPs lack ligand specificities but could be easily produced *in vitro* and crystallized due to their small size and solubility, allowing a ligand screening of potential odorant disruptants, in combination with molecular docking analysis and dynamic stimulations. In contrast, ORs present complex crystallization procedures, however, they generally are more specific to odorants, which indicates that disrupting ORs could interfere with specific behaviors of insects (Venthur and Zhou). With a deeper understanding of the molecular mechanisms of insect olfaction and technological advances (especially on protein 3-D modeling and ligand docking, gene editing and manipulation), we will witness breakthrough achievements on the application of olfaction-based techniques in control of pests and vector insects in the near future.

## Author Contributions

PH, ND, and S-LD wrote, edited, and finalized this manuscript.

### Conflict of Interest

The authors declare that the research was conducted in the absence of any commercial or financial relationships that could be construed as a potential conflict of interest.

